# Efficiency of bulk-heterojunction organic solar cells

**DOI:** 10.1016/j.progpolymsci.2013.05.001

**Published:** 2013-12

**Authors:** M.C. Scharber, N.S. Sariciftci

**Affiliations:** Linz Institute of Organic Solar Cells (LIOS), Physical Chemistry, Johannes Kepler University Linz, Altenbergerstrasse 69, 4040 Linz, Austria

**Keywords:** Organic solar cell, Bulk heterojunction solar cell, Power conversion efficiency

## Abstract

During the last years the performance of bulk heterojunction solar cells has been improved significantly. For a large-scale application of this technology further improvements are required. This article reviews the basic working principles and the state of the art device design of bulk heterojunction solar cells. The importance of high power conversion efficiencies for the commercial exploitation is outlined and different efficiency models for bulk heterojunction solar cells are discussed. Assuming state of the art materials and device architectures several models predict power conversion efficiencies in the range of 10–15%. A more general approach assuming device operation close to the Shockley–Queisser-limit leads to even higher efficiencies. Bulk heterojunction devices exhibiting only radiative recombination of charge carriers could be as efficient as ideal inorganic photovoltaic devices.

## 1 Introduction

Thin film photovoltaic cells based on solution processable organic semiconductors have attracted remarkable interest as a possible alternative to conventional, inorganic photovoltaic technologies. The following key advantages of organic photovoltaic (OPV) devices have been identified:

1. Low weight and flexibility of the PV modules.
2. Semitransparency.
3. Easy integration into other products.
4. New market opportunities, e.g. wearable PV.
5. Significantly lower manufacturing costs compared to conventional inorganic technologies.
6. Manufacturing of OPV in a continuous process using state of the art printing tools.
7. Short energy payback times and low environmental impact during manufacturing and operations.

**Fig. 1:**
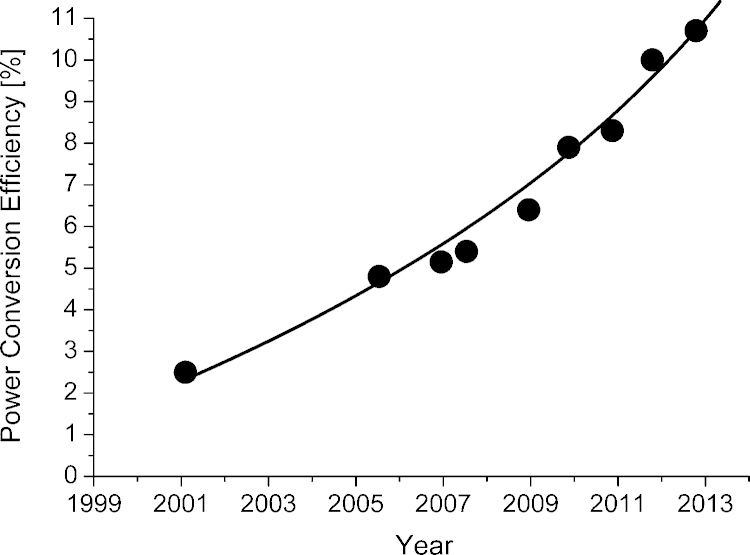
Certified record power conversion efficiencies of single junction organic solar cells published in Progress in Photovoltaics. The first point in the graph (year 2001) is not listed in any efficiency table.

Most of the advantages listed above do also apply to solar cells based on vapor-deposited small molecule absorbers. This suggests that OPV does have the potential to be a disruptive technology within the PV market. The bright outlook has initiated a lot of research and development activities and substantial progress has been made in increasing the power conversion efficiency (PCE) of solution processed OPV during the last years. In 2001 Shaheen et al. [1] reported a record efficiency of 2.5%. About 10 years later, Mitsubishi Chemical demonstrated a PCE > 10% for lab devices with an active area of ∼1 cm^2^ [2]. In Fig. 1 the development of the power conversion efficiency of bulk heterojunction solar cells is summarized. Device data were taken from the solar cell efficiency tables published in Progress in Photovoltaics: Research and Applications [3], [4], [5], [6], [7], [8], [9]. In addition, several different printing and coating processes have been developed and classical roll-to-roll processing of organic solar cells has been demonstrated. Today fully printed prototypes are manufactured and first products are available [10]. Commercially available modules do show power conversion efficiencies in the range of 1.5–2.5%.

Remarkable improvement in durability of bulk-heterojunction solar cells remarkable progress has been achieved during the last ten years. While the first devices had to be stored in an inert atmosphere, and degraded quickly on exposure to sunlight, today small organic PV modules on flexible substrates with operational lifetimes of a few years are available [11]. Jorgensen et al. summarized the status of the current understanding of organic photovoltaic stability in a recently published review article [12]. The initiative “International Summits on Organic Photovoltaic Stability” (ISOS) has supported efforts to investigate and improve OPV stability by establishing standard testing protocols and initiating focused research efforts on organic solar cell degradation. Although several studies indicate that a clever design of photoactive materials will improve the photochemical stability of organic absorber layers, rigorous encapsulation appears to be mandatory to ensure long term stability. Manceau et al. [13] investigated the photochemical stability of semiconducting organic polymers under simulated sunlight and ambient atmosphere. They found that the molecular structure including the attached side chains have a strong impact on the photochemical stability. Based on their results they suggest guidelines for the design of polymers with an improved photochemical stability in the presence of ambient air. Tromholt et al. [14] and Hoke et al. [15] studied the photochemical stability of organic semiconducting donor acceptor blends under illumination and ambient conditions. They found that the electron affinity of the acceptor determines the degradation rate of the semiconductor layer.

Besides the detailed understanding of photochemical processes in organic absorber layers, the development of alternative device designs has resulted in a significant improvement of the device stability. In early devices low work function metals like calcium, barium or aluminum were applied as cathodes. Exposure to oxygen or water caused an almost immediate oxidation of these electrodes resulting in a fast degradation of the power conversion efficiency. By introducing the so-called inverted design [16] this fast electrode degradation could be overcome [17]. The low work function metals has been replaced by transparent oxides like zinc oxide [17] or titanium dioxide [16] or the work function of stable electrode materials has been modified using a thin interfacial layer [18] to form the cathode of the solar cell. As anode materials sliver gold or standard hole-injection layers like PEDOT:PSS has been applied. An additional advantage of the inverted device design is that all layers can be deposited from solution and no vacuum process is required [17].

Overall there has been remarkable progress in the field of organic solar cells. Within less than 20 years double digit efficiencies and reasonable lifetimes were achieved. However, before large scale commercialization and entering a direct competition with state of the art inorganic PV technologies, further improvements especially in the power conversion efficiency are required. The review is organized as follows: after the introduction the device architecture and the working principle of bulk heterojunction solar cells are summarized. In the third section we discuss the importance of the power conversion efficiency (PCE) of photovoltaic cells for large area electricity production. In Section 4 we describe a general efficiency limit of photovoltaic cell followed by models for the efficiency of bulk-heterojunction solar cells and a short summary.

## 2 Bulk heterojunction solar cell basics

**Fig. 2:**
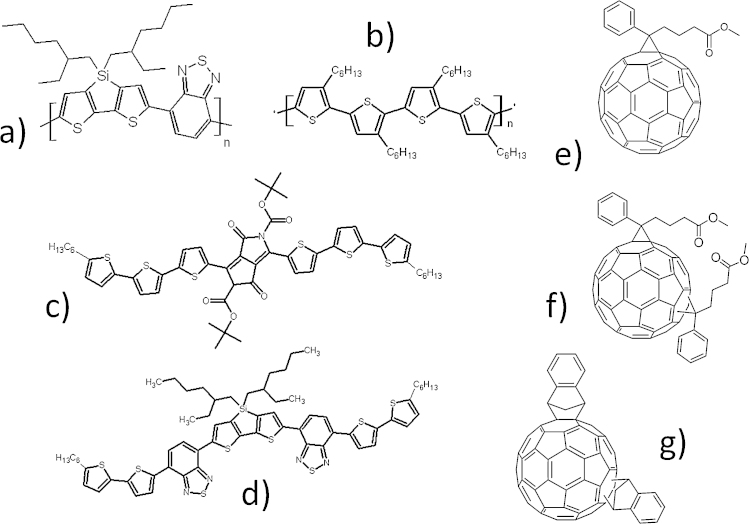
Examples for donor and acceptor materials used in bulk heterojunction solar cells. (a) Poly[(4,4′-bis(2-ethylhexyl)dithieno[3,2-b:2′,3′-d]silole)-2,6-diyl-alt-(4,7-bis(2-thienyl)-2,1,3-benzothiadia-zole)-5,5′-diyl] [20], (b) poly-(3-hexylthiophene-2,5-diol), (c) diketopyrrolopyrrole based oligomer [21], (d) 5,5′-bis{(4-(7-hexylthiophen-2-yl)thiophen-2-yl)-[1,2,5]thiadiazolo[3,4-c]pyridine}-3,3′-di-2-ethylhexylsilylene-2,2′-bithiophene, (e) [6,6]-phenyl-C_61_-butyric acid methyl [22], (f) bisadduct analog [6,6]-phenyl-C_61_-butyric acid methyl and (g) indene-C_60_-bisadduct.

The absorber layer of an efficient state of the art bulk heterojunction solar cell is made of so-called donor and acceptor molecules. As donors usually conjugated polymers, oligomers or conjugated pigments, as acceptors frequently fullerene derivatives are applied (Fig. 2). Often these materials are classified as organic semiconductors [19]. They are known for their outstanding optical properties and their ability to transport charges [19].

**Fig. 3:**
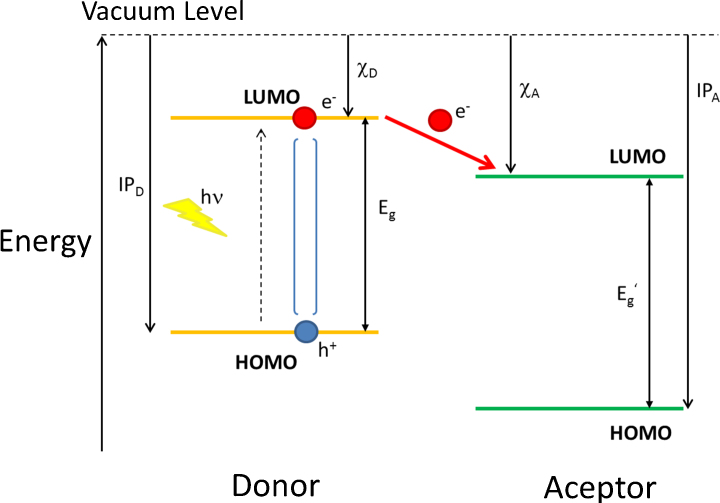
Energy level diagram of a donor acceptor system; IP is the ionization potentials, is the electron affinity. The arrow between the LUMO-levels indicates the photoinduced electron transfer which is the first step for generating free charge carriers.

A schematic diagram of the energy levels of a typical donor acceptor system is shown in Fig. 3. HOMO denotes the highest occupied molecular orbital and LUMO the lowest unoccupied molecular orbital of the organic molecules. It is generally accepted that in state of the art organic semiconductors a bound electron–hole pair (exciton) is generated upon photon absorption [23]. Due to the low dielectric constant of organic materials, there is a strong Coulomb attraction between the electron and the hole and a dissociation of the exciton into free charges is very unlikely under ambient conditions. Considering an oversimplified model of an electron–hole pair separated by 1 nm in a material with a dielectric number of 3–4, applying Coulomb law results in an electron–hole binding energy in the range of 0.35–0.5 eV. This binding energy exceeds the thermal energy at room temperature by an order of magnitude and electron acceptor molecules need to be added to an organic semiconductor donor to facilitate the generation of free charge carriers. The difference in the lowest unoccupied molecular orbital energies or electron affinities of the donor and acceptor material creates the driving force for the rapid transfer of an electron from the donor to the acceptor [24]. Due to the relatively short (<1 ns) exciton lifetime in organic semiconductors—quantitative charge generation requires very fast charge separation. For prototype systems charge generation times below 100 fs have been found [25]. Upon photo-excitation of the acceptor moiety charge carriers can be generated via hole-transfer from the acceptor to the donor [26]. This means that both, the donor and the acceptor can contribute to the action spectrum of the solar cell.

**Fig. 4:**
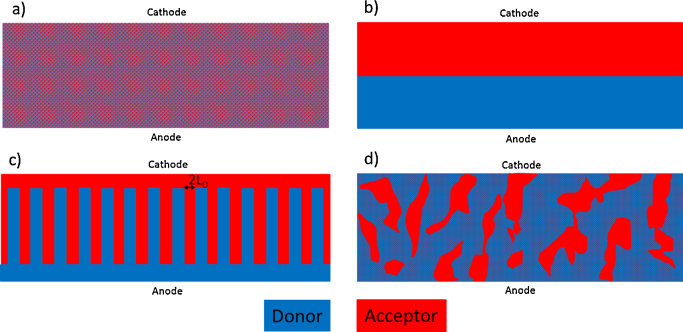
Schematic cross-section of nanomorphologies of bulk heterojunction solar cells. (a) Fine mixture of donor and acceptor molecules, (b) bilayer arrangement, (c) ideal morphology of a bulk heterojunction solar cells and (d) typical morphology of a solution processes device.

A second prerequisite for efficient charge generation is that excitons are generated within their diffusion length *L*_D_ to the nearest donor–acceptor interface. Recent measurements [27] indicate that *L*_D_ is in the range of 10 nm for several prototype conjugated polymers used in bulk heterojunction solar cells, which means that an intermixing of the donor and the acceptor moieties on the nanometer scale is required. This insight lead to the so-called bulk-heterojunction concept which was reported by Yu et al. [28] for conjugated polymer based solar cells in 1995. In their manuscript the author showed that by blending the donor (poly(2-methoxy-5-(2′-ethyl-hexyloxy)-1,4-phenylene vinylene)) (MEH-PPV) and acceptor (Fullerene C_60_) molecules in the photoactive layer, the incident photon to electron conversion efficiency increased 10 fold compared to conventional donor–acceptor bilayer devices. The authors attributed the observed performance increase to the large interfacial area between the donor and the acceptor material in their so-called interpenetrating phase-separated donor–acceptor network composite. They also highlighted the importance of controlling the nano-morphology of the donor–acceptor blend which has been one of the dominate research areas during the last years [29], [30]. There is still no full consensus how the ideal nano-morphology of a bulk heterojunction should look like. A very fine dispersion of the acceptor in the donor material (Fig. 4a) will lead to efficient charge generation but poor charge transport. Ideal charge transport could be achieved by arranging the donor and acceptor in by bilayer stack (Fig. 4b). On the other hand, charge generation happens only at the interface between the two layers and will be overall poor. Calculations and morphology simulation work have suggested that the arrangement shown in Fig. 4c should lead to ideal performance [31]. Highly ordered donor and acceptor domains will ensure excellent charge transport. A domains width of 2 times the exciton diffusion length will facilitate efficient charge generation at the same time. This nano-patterned morphology is very difficult to prepare and device manufacturing often does rely on phase separation of the donor and acceptor materials during the formation of the absorber film. A cross section of “real” bulk-heterojunction solar cell is sketched in Fig. 4d. Connected domains with a typical size of several tens of nanometers are formed in the film. At the same time a small amount of the acceptor material may be dissolved in the donor domains or vice versa [32], [33], [34]. Several methods are available today to optimize the interfacial area between the donor acceptor phases. For some materials an additional thermal treatment [35] of the photoactive layer was found to be beneficial. Also the proper selection of processing solvents or the use of processing additives [36] (e.g. diiodooctane or dithiols) resulted in improved donor–acceptor arrangements.

Overall the nano-morphology has been found to be very important when it comes to the efficiency optimization of a given material combination. Detailed reviews on nano-morphology studies of OPVs are available elsewhere [37]. However, the nano-morphology does have little or no impact on the ultimate efficiency limit of organic solar cells. As discussed below, the required difference in the electron affinities of the donor and acceptor moieties causes such an intrinsic loss which is not present in conventional inorganic solar cells. In most of the models the power conversion efficiency of organic solar cells not only depends on the optical bandgap but also on difference of the electron affinities of the donor an acceptor moieties.

**Fig. 5:**
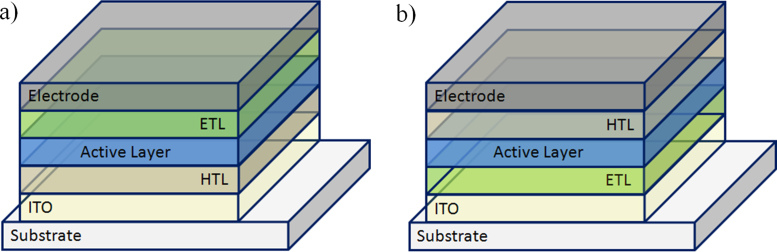
Different device architectures of bulk heterojunction solar cells. (a) Standard device design with the cathode on top of the device stack and (b) inverted device architecture with the cathode located on the transparent substrate.

Often bulk heterojunction solar cells are built on a transparent substrate coated with a conductive and transparent electrode material. Due to its excellent transparency and conductivity ITO (indium tin oxide) is applied in most of the reported devices. In the so called standard configuration (Fig. 5a) the ITO is coated with a hole transport layer (HTL). Thin films of doped conjugated polymer like PEDOT:PSS (poly(3,4-ethylenedioxythiophene) poly(styrenesulfonate)) or a thin oxide layer (e.g. MoO_3_) have been used as HTLs. On top of the HTL the photoactive layer, a blend of donor and acceptor material is coated followed by an optional electron transport layer (ETL) and a low work function electrode. ETL materials are often oxides like zinc oxide or titanium dioxide. Such a transparent thin layer can also improve the light absorption in the photoactive layer when used as a so-called optical spacer [38]. In the standard architecture, the top electrode is the cathode and calcium, barium or aluminum are applied for collecting the electrons generated in the photoactive layer.

In the inverted architecture the transparent electrode coated on the substrate acts as a cathode. Either by modifying the work function of the electrode material, by applying an interfacial layer [18] or by using transparent oxides like zinc oxide [39] or titanium dioxide [40] a selective contact to the acceptor material in the active layer is formed. On top of the active layer a HTL like PEDOT:PSS or a thin oxide layer (e.g. MoO_3_) has been used and the device is finalized with an air stable high work function electrode material like silver or gold. Both device architectures allow the preparation of high performance bulk heterojunction solar cells [16]. The inverted design offers processing advantages (no vacuum process is required) and shows improved ambient stability due to the absence of a low work function electrode.

## 3 Toward high power conversion efficiencies

Over the years the cost of electricity produced by PV could be reduced significantly. While the levelized cost of PV electricity was about 0.5 €/kWh in Germany in 2007, PV electricity reached the private household price level of approximately 0.25 €/kWh at the end of 2011 [41]. This is often referred to that solar electricity has reached the grid parity for private household electricity. A strong reduction of the costs of PV-modules and inverters has led to this positive development. Nevertheless, the production costs of electricity generated by PV are still significantly higher compared to electricity produced by conventional power plants. According the EIA Annual Energy Outlook 2011 [42] hydro-, coal- or gas-powered plants can produce electricity well below 0.1 €/kWh. This means that for making a significant contribution to the global electricity or energy production the costs for solar electricity needs to undergo substantial further cost reductions.

Two approaches have been discussed to achieve cheaper PV-electricity [43]:

(a) Increasing the power conversion efficiency while keeping PV-material costs the same (wafer-based solar cells (1st generation) or high efficiency concepts (3rd generation)).
(b) Developing low-cost, moderate efficiency PV-material (thin-film PV, 2nd generation).

Both approaches have been pursued and wafer-based silicon PV and thin-film based CdTe solar cells appear to be the most promising/competitive technologies in today's PV markets.

Analyzing the typical cost structure of a grid connected PV-system one can identify three main contributions:

(a) PV module.
(b) Installation/balance of system.
(c) Power electronics (inverter).

Recently the Department of Energy published several reports providing an in-depth assessment of the potential of solar technologies [44], [45]. In a white paper [44] an installed utility-scale PV system price of 3.4 US$/W is reported for the year 2010. The authors expect utility scale system costs to reach 2.2 US$/W by 2016 (if not sooner) and they raise the 1 US$/W goal which would make solar electricity competitive without additional subsidies at a wholesale rate of electricity nearly everywhere in the US. The PV-system cost structures for 2010, 2016 and the 1 US$/W goal determined by the DOE are summarized in Table 1.

**Table 1:** PV-system cost – Department of Energy (DOE).

	2010 US$/W	2016 US$/W	1 US$/W US$/W
Module	1.7 (50%)	1.05 (48%)	0.5 (50%)
Installation	1.48 (44%)	0.97 (44%)	0.4 (40%)
Power electronics	0.22 (6%)	0.18 (8%)	0.1 (10%)

As shown in Table 1, about 50% of the costs are related to the PV modules. Power Electronics contribute 10% and Installation about 40% to the overall costs. While module costs are proportional to the installed peak power, the installation costs scale with the installed area. This means that PV-system based on low efficiency modules will have higher costs for installation compared to systems comprising high efficiency PV material. Assuming that the installation costs per area are about the same for the different technologies, PV modules with half the efficiency of the state-of the art modules will not be able to compete. This finding is illustrated in Table 2. Assuming the DOE 2010 cost structure (Table 1), system costs of 2000 US$/kW and a module efficiency of 15%, one finds about 880 US$/kW for the installation costs.

**Table 2:** PV system cost scenarios for different system efficiencies.

Cost ($)	Efficiency (%)	Area (m^2^)	Module	Inverter	Installation
			50%	6%	44%
2000	15.00	6.67	1000	120	880
2000	10.00	10.00	561	120	1319
2000	5.00	20.00	−759	120	2639
2000	7.00	14.29	−5	120	1885

With the same installation costs per m^2^ the installation of a system based on 10% efficient modules will cost about 1319 US$ per installed kW. When using the same power electronics, about 561 Euro can be spent for the lower efficiency modules to reach the same system costs. At a power conversion efficiency of about 7% the cost for inverter and installation add up to the costs of the initial system and the PV modules would have to be for free to compete with the PV installation based on the 15% efficient PV generators. For the future DOE scenarios (Table 1) the situation is very similar. As the overall cost structure is about the same a minimum efficiency of 7–8% is necessary to compete with 15% efficient PV modules. However, at this efficiency the modules need to be free of charge.

The cost considerations above may be oversimplified and the derived minimum efficiency does depend on various assumptions. Therefore the given numbers should only be indicative and the discussion should illustrate the importance of the area dependent costs of a photovoltaic system. The calculations show that PV technologies focusing on ultra-low cost and accepting low power conversion efficiencies do have a narrow commercialization window and that substantially lower installation costs may be required when applying low efficiency solar cells. Due to the inferior power density, low efficiency PV will not be the prefer choice when the area for collecting solar radiation is limited (e.g. roof-top), which may be an additional argument for the high efficiency approach. On the other hand the installation of flexible and low weight PV-modules could be much simpler compared to state-of-the-art modules, which should also result in lower installation costs. To which extend this could increase the competitiveness of low efficiency PV-material needs to be seen.

## 4 Photovoltaic efficiency limits

Photovoltaic cells are often associated with quite modest solar radiation to electricity conversion efficiencies. State of the art commercial solar modules usually show efficiencies in the range of 10–20%. However, considering thermodynamic arguments solar radiation to electricity conversion efficiencies close to the Carnot efficiency of 95% may be achievable. This value is given by the limit for converting heat in a Carnot engine into, e.g. mechanical energy applying a heat source with a temperature of 5800 K (surface of the sun) and a heat sink at 300 K. Even under more realistic conditions allowing the generation of entropy during the conversion process very high efficiencies are conceivable. As discussed by Würfel [46] a system with an efficiency potential of up to 85% can be constructed based on a black body absorber. For state of the art single band-gap semiconductor based photovoltaic cells the maximum solar radiation to electricity conversion efficiency is smaller. In 1961 the “Detailed Balance Limit of Efficiency of p–n Junction Solar Cells” by William Shockley and Hans J. Queisser was published [47]. Based on an analysis of the microscopic radiative processes in a pn-solar cell, they derived the maximum efficiency and the current–voltage curve of an ideal photovoltaic cell. Assuming a light source corresponding to a 6000 K black body radiator and the same solid angle of appearance subtended by the sun, Shockley and Queisser derive a maximum efficiency of 30% and an optimum absorber band gap of 1.1 eV.

**Fig. 6:**
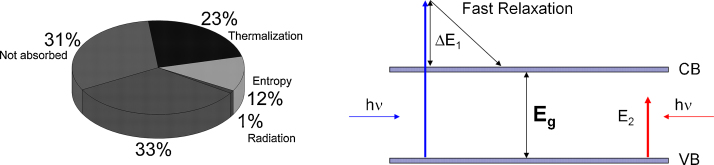
Illustration of the different losses in an ideal solar cell, photon energy larger or smaller the absorber gap (left); power conversion efficiency losses according to the Shockley–Queisser analysis. Δ*E*_1_ is the photon access energy which is lost due to fast relaxation of the photoexcited charge carrier. Photons with energy *E*_2_ smaller than the band gap are not absorbed by the semiconductor.

Later on several manuscripts [48], [49], [50] discussing the ultimate limit for the conversion of solar energy were published. Often thermodynamic arguments were used in the performed calculations. Despite the different approaches all of them confirm the earlier results by Shockley and Queisser. The small variations in the maximum efficiency and optimum absorber band gap stem often from different solar spectra used for the calculations. Archer and Bolton found a limiting conversion efficiency of ∼33% at the optimal bandgap of 1.34 eV assuming air mass 1.5 illumination [49]. Their analysis reveals that about 31% of the light energy is lost because the photon energy is too small to excite the absorber (*E* < *E*_g_), about 23% of the energy is lost due to intraband thermalization of photo-excited charge carriers, about 12% origin from entropic losses and 1% of the energy is lost via radiative recombination. The different losses are illustrated and summarized in Fig. 6.

For their analysis Shockley and Queisser applied the following assumptions:

1. Each absorbed photon generates one electron hole pair.
2. Perfect charge collection in the solar cell.
3. Radiative recombination as the only recombination process of charge carriers.

The first two assumption ensure that each absorbed photon in converted into a collectable charge carrier. Assuming only radiative recombination leads to the maximum lifetime of photo-generated charge carriers and to the largest charge carrier concentration when a device is operated under open circuit conditions. This results in the largest splitting of the quasi Fermi-levels and the highest achievable open circuit voltage. Any non-radiative recombination channel will reduce the charge carrier lifetime and by that the open circuit voltage of the solar cell. As a consequence, the photoluminescence decay of the radiative species involved in the generation and recombination process of charge carrier in a solar, measured under open circuit conditions needs to show the same kinetics then the decaying charge carriers.

**Fig. 7:**
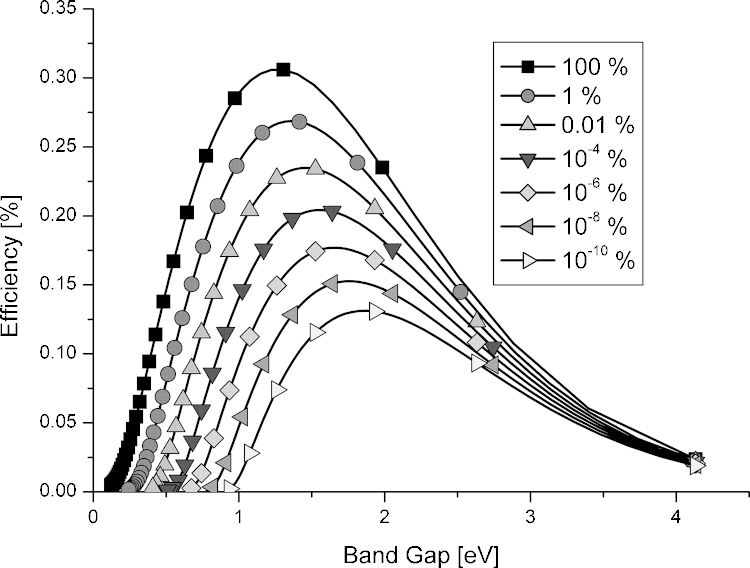
Shockely–Queisser efficiency limit for solar cells with different radiative recombination contributions (from 100% down to 10^−10^% radiative recombination) [53].

The importance of the radiative decay for the performance of solar cells has been discussed earlier [51], [52]. An analysis by Green shows that even the most efficient small area devices operate relatively far away from the radiative limit [53]. Applying the “Reciprocity relation between photovoltaic quantum efficiency and electroluminescent emission of solar cells” [54] he determines the so-called external radiative efficiency (ERE), which is defined as the fraction of the total dark current recombination in the device that results in radiative emission from the device when operated under open circuit conditions. The ERE is a measure for the non-radiative recombination active in a solar cell and Green finds an ERE of ∼0.5% for crystalline silicon solar cells and much smaller values for the best dye-sensitized, cadmium telluride, amorphous silicon and organic solar cells. Only for highly efficient gallium arsenide devices EREs > 1% were found. Also in the original manuscript by Shockely and Queisser the effect of non-radiative recombination is discussed. In Fig. 7 the dependence of the efficiency-band gap relation as a function of the radiative recombination coefficient is shown. If there is only radiative recombination the maximum efficiency is about 33%. If only 0.01% of the charge carriers recombine radiatively an efficiency of 23% can be reached. Even at very low radiative recombination rates (10^−6^ to 10^−8^%) power conversion efficiencies in the range of 15–18% are feasible.

The considerations above show that low emission absorber layers can give reasonable power conversion efficiencies in photovoltaic cells. This is of particular importance for organic solar cells. They are based on an efficient separation of highly emissive excitons and one indication for an efficient charge generation is the complete quenching of the excitonic emission. As the recombination of free charges is also predominantly non-radiative in state of the art devices (ERE ∼ 10^−7^%) [53]—one would expect a maximum efficiency in the range of 15–16% according to the work of Shockely and Queisser (Fig. 7).

## 5 Models for bulk heterojunction solar cell efficiencies

There are several microscopic descriptions available modeling the optical and electrical processes in bulk heterojunction solar cells [55], [56]. Those models give a detailed insight into the performance limiting processes. On the other hand they are based on a large set of parameters and assumptions and a deduction of the ultimate efficiency through those models is often very difficult. To calculate the power conversion efficiency of an actual solar cell the so-called open circuit voltage, the short circuit current and the electrical fill factor need to be determined. The efficiency of a solar cell is given as the fraction of incident power which is converted to electricity and is defined as the open circuit voltage (*V*_oc_) times the short circuit current (*I*_sc_) times the electrical fill factor (FF).The open-circuit voltage is the maximum voltage available from a solar cell, and this occurs when no current is flowing through the solar cell. The short-circuit current is the current through the solar cell when the voltage across the solar cell is zero (i.e., when the solar cell is short circuited). The FF is defined as the ratio of the maximum power from the solar cell to the product of *V*_oc_ and *I*_sc_.

Below we will review “empirical” efficiency models based on experimental observations and a small set of assumption. After that we summarize efficiency models following the Shockely–Queisser approach, discuss the role of the charge transfer complex and briefly cover a thermodynamic description of the organic solar cell efficiency.

### 5.1 Empirical models

**Fig. 8:**
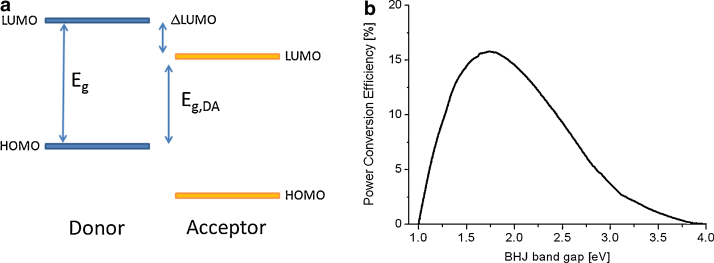
(a) Schematic diagram of the energetic levels of a donor acceptor system. *E*_g,DA_ is often called the effective band gap and (b) power conversion efficiency versus the absorber band gap derived assuming a loss of 1 eV per thermalized charge carrier [57].

In 2004 Coakley and McGehee [57] analyzed the performance potential of bulk heterojunction solar cell. A very simple model was applied to estimate the ultimate power conversion efficiency. Assuming all photons with an energy larger than the band gap energy are absorbed and each photo-generated electron stores the band gap energy minus the losses occurring during the charge transfer (ΔLUMO). The maximum theoretical efficiency that can be derived as a function of the band gap of a conjugated polymer, when electrons loose 1 eV during electron transfer to an electron acceptor, is shown in Fig. 8.

With this assumption a maximum power conversion efficiency of ∼15% is found with an ideal absorber band gap of 1.75 eV.

Based on an empirical relation for the open circuit voltage, Scharber et al. derived “Design Rules for Donors in Bulk-Heterojunction Solar Cells” and concluded that 10% energy-conversion efficiencies are achievable for donor–acceptor organic solar cells [58]. A systematic study on a large set of donor polymers combined with the acceptor molecule PCBM ([6,6]-phenyl-C_61_-butyric acid methyl) revealed that the open circuit voltage of optimized solar cells can be empirically calculated by

(1) $$V_{\text{oc}}-\frac{1}{e}(E_{\text{HOMO}}^{\text{DONOR}}-E_{\text{LUMO}}^{\text{Acceptor}})-0.3\;V$$

Here *e* is the elementary charge and 0.3 V is a typical loss found in bulk heterojunction solar cells. The energies of the donor HOMO-level and the acceptor LUMO-level are given in electron volts. A similar relation has been reported by Veldman et al. [59] based on a detailed analysis of the charge transfer emission in polymer fullerene blends. A number of studies suggested the physical reasons of this 0.3 V loss indicated in Eq. (1) underlining the effect of disorder to the maximum achievable open circuit voltage in organic solar cells [60], [61]. Durrant and coworkers [62], [63] highlighted the impact of charge carrier recombination and the microstructure of the donor acceptor blend on the open circuit voltage of bulk heterojunction solar cells. Based on transient optoelectronic analyses they developed a comprehensive model describing the open circuit voltage of BHJ devices. They found for different polymer-fullerene solar cells open circuit voltage losses in the range of 0.225–0.435 V.

**Fig. 9:**
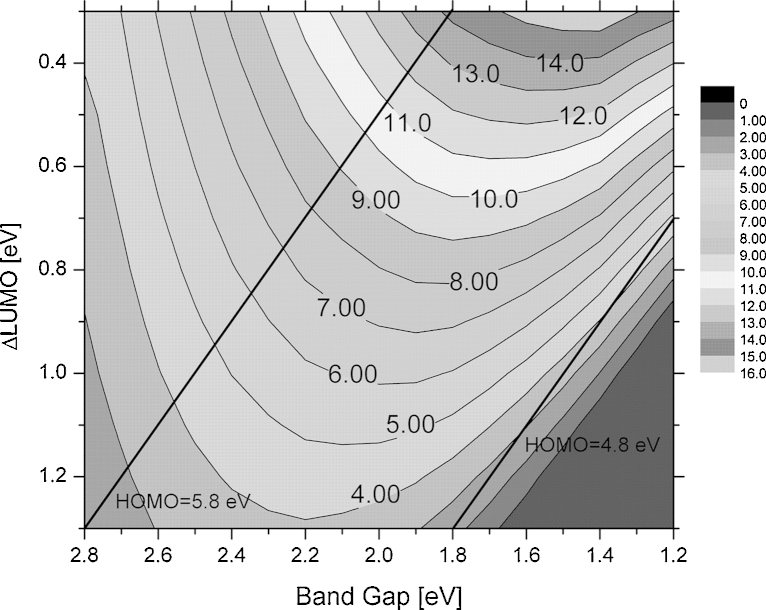
Contour plot showing the power conversion efficiency of a bulk heterojunction solar cell with PCBM as acceptor material (LUMO level 4.3 eV). For the calculation an EQE of 80%, a FF of 75% and an open circuit voltage according to Eq. (1) was used. Black lines indicate constant HOMO levels of 5.8 eV and 4.8 eV respectively.

Assuming typical external quantum efficiencies and electrical fill factors, Eq. (1) can be used to calculate the power conversion efficiency as a function of the solar cell band gap and the LUMO position. In 2006 the highest reported external quantum efficiencies (EQEs) and fill factors (FFs) were in the range of 65%. The EQE is defined as the probability that an incident photon is converted into a charge carrier which is collected at an electrode of the solar cell. With a minimum ΔLUMO of 0.3 V (to ensure efficient charge transfer), a maximum efficiency of 11% was derived and the optimum band gap was found to be 1.45 eV. Today larger FFs and EQEs have been reported for highly efficient OPV cells and in Fig. 9 the power conversion efficiency of bulk heterojunction solar cells is plotted as a function of the absorber band gap and the LUMO-level offset assuming an EQE of 80% and a fill factor or 75%.

The maximum efficiency increases from 11% (EQE and FF at 65%) to about 15%. The optimal gap is unchanged at 1.45 eV. The contour plot and the underlying model have been used extensively to evaluate the potential or new organic semiconductors [64], [65], [66]. The proposed design rules have been the basis for computer based material design approaches [65]. Despite its simplicity, the model is still valid today and all high performance materials show efficiencies within the available contour plots.

Minnaert and Burgelman [67] developed a realistic optical absorbance model for OPV by introducing a finite bandwidth of the photoactive layer and assuming an open circuit voltage given by the donor-HOMO minus the acceptor-LUMO difference times a so-called voltage factor *f*. With a ΔLUMO of 0.3 eV they calculate maximum efficiencies in the range of 5–15% depending on the spectral width of the absorption window of the organic semiconducting absorber blend, the voltage factor, the electrical fill factor and the external quantum efficiency. Overall the efficiencies derived by Minnaert at al. are very similar to the one found by Coakley and Scharber illustrating that the finite absorber bandwidth is not detrimental for achieving high solar cell performance.

In 2006 Blom and coworkers calculated the ultimate efficiency of polymer-fullerene bulk heterojunction solar cells [68] by using an advanced drift-diffusion device model [69]. They started by simulating a state of the art bulk heterojunction based on a poly-3-hexyl-thiophene – PCBM absorber to obtain typical bulk-heterojunction solar cell device parameters. Using those parameters the authors optimized the LUMO-level offset, the layer thickness and the charge carrier mobility and they found an ultimate power conversion efficiency of 10.8% for a 200 nm thick layer and an optimum band gap of 1.9 eV.

In 2009 Marks and coworkers developed a practical efficiency limit of organic photovoltaic cells [70]. They introduced an exciton dissociation factor representing the exciton dissociation field dependence, which has an impact on the electrical fill factor. By applying Green's method [71] an equation for the electrical fill factor was derived. Using Eq. (1) and varying the ΔLUMO a practical efficiency limit of ∼14% was found when an electric field independent charge generation process was assumed.

In summary, the empirical models discussed here suggest that efficiencies in the range of 10–15% can be achieved by optimizing the LUMO-LUMO offset of the donor–acceptor system and the band gap of the solar cell. In Table 3 the results including the optimum gap are summarized.

**Table 3:** Summary estimates of ultimate power conversion efficiency of organic donor acceptor solar cells.

Author	Efficiency (%)	Optimum band gap (eV)
Coakley et al. [25]	15	1.75
Scharber et al. [26]	11–15	1.45
Minnaert et al. [27]	5–15	1.5
Koster et al. [28]	11	1.9
Servaites et al. [30]	14	1.6

There is still no agreement on the ideal band gap for bulk heterojucntion solar cells. The organic solar cells and sub-modules listed in the most recent progress in photovoltaics efficiency table [9] are based on absorbers with band gaps in the range of 1.4–1.6 eV reaching efficiencies up to 10%.

Recently Koster et al. [72] reported the effect of increasing the dielectric constant *ɛ* of the absorber material on the power conversion efficiency of organic solar cells. A larger *ɛ* leads to a smaller exciton binding energy and a smaller LUMO-LUMO offset would be sufficient for quantitative free charge carrier generation. Their model suggests that by increasing *ɛ* for 3–8, the power conversion efficiency would increase for ∼12% to about 20%. At a dielectric constant of 8, the exciton binding energy would be in the range of 25 meV which would allow the generation of free charge carriers via thermal dissociation.

### 5.2 Charge transfer complex and detailed balance limit for OPV

A detailed study by Veldman et al. [59] on the charge transfer complex in bulk heterojunction solar cell revealed that the minimum open circuit loss amounts to 0.6 eV compared to the lower bandgap either of the donor and the acceptor. They also observe a linear relation between the open circuit voltage and the HOMO–LUMO difference of the donor acceptor pair and the *V*_oc_ and the energetic position of the charge transfer complex emission. With this relation for the open circuit voltage and assuming values for the EQE and the FF they calculated the ultimate efficiency of BHJ cells. With a FF and EQE of 65% they find a maximum efficiency of 11% at a band gap around 1.4 eV. Veldman's work illustrates the importance of the charge transfer complex in organic solar cells. Below we will discuss an efficiency model focusing on the nature of the charge transfer complex in more detail.

In 2009 Kirchartz et al. [73] calculated the radiative efficiency limit of organic solar cells. Adapting the Shockely–Queisser approach and based on the optical properties of a state of the art polymer fullerene blend materials (PF10TBT/PCBM) they estimate a maximum efficiency >20% for pristine polymer and polymer fullerene absorbers. As the devices the analysis was based on showed a power conversion efficiency of max. 4.2%, the authors analyzed the losses leading to this tremendous PCE reduction. They found that

1. Optical losses.
2. Exciton losses due to insufficient transport of excitons to the next donor–acceptor interface or due to inefficient exciton dissociation.
3. Non-radiative recombination losses.
4. Charge carrier collection losses due to insufficient mobilities.

The by far most dominant loss was attributed to non-radiative recombination at the donor–acceptor interface. The work by Kirchartz et al. [73] shows the dilemma of state of the art bulk heterojunction solar cells. On the one hand all generated excitons should be dissociated at a donor–acceptor interface, which does lead to a strong quenching of the photoluminescence (PL). Therefore PL-quenching experiments are frequently used to evaluate the potential of novel organic solar cells as absorbers in BHJ devices. On the other hand a solar cell operating close to the Shockely–Queisser limit should exhibit only radiative recombination meaning that under open circuit conditions, the photoluminescence quantum yield should be 100%. Today's state of the art absorber layers do show a weak emission resulting from a charge transfer state recombination. This charge transfer state has been extensively investigated by Vandewal et al. [74], [75]. They analyzed the organic solar cells based on a detailed balance theory that adds the aspect of electronic transport to the SQ theory. This reciprocity relations between photovoltaic quantum efficiency and electroluminescent emission of solar cells and a second relation between the external quantum efficiency of the solar cell when operated as LED with the open circuit voltage *V*_oc_ of a solar cell initially developed Rau were applied to several different bulk-heterojunction solar cells and the authors found good correlations when considering the charge transfer emission.

The finding that the charge transfer state could be the emissive species to be considered within the detailed balance limit triggered further activities to estimate the ultimate power conversion efficiency. Vandewal et al. [75] analyzed the charge transfer state in three different organic semiconductor absorbers and determined the radiative limit following an approach similar to the one applied be Kirchartz et al. [73]. They found for an organic solar cell with the low gap absorber layer PCPDTBT:PC_71_BM an ultimate power conversion efficiency of ∼28%. If the very low radiative recombination quantum yield (∼10^−6^) is included into the calculation, the ultimate efficiency drops to about 16%. Best measured efficiencies for this absorber system are in the range of 6% [36].This example shows that under idealized conditions, organic solar cells could be as efficient as their inorganic counter parts. However, today all state of the art absorbers are far away from ideal performance and novel materials will be necessary for approaching the Shockely–Queisser Limit.

**Fig. 10:**
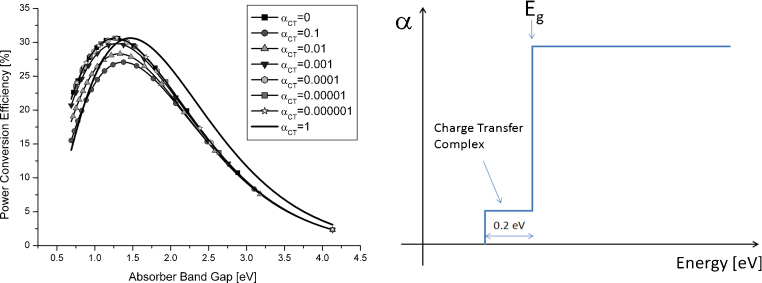
(a) Profile of an ideal absorber with a weak and 0.2 eV broad charge transfer absorption feature. (b) Power conversion efficiency of a solar cell with a absorption profile (a) and different absorptions strength (CT) of the charge transfer state [68], [76].

Recently Koster et al. [72] and Gruber et al. [76] pointed out that either a very weakly absorbing or a strongly absorbing charge transfer state does lead to the highest power conversion efficiencies under the radiative efficiency limit. This suggests that the charge transfer state par-se is not essential for highest power conversion efficiencies. A weakly absorbing CT does minimize the losses in *V*_oc_ while a strongly absorbing charge transfer state will allow the collection of additional solar photons. In both cases, the ultimate efficiency in the radiative limit is above 30%. The situation is illustrated in Fig. 10. Here we assume a charge transfer state with different absorptions strength with a constant spectral width of 0.2 eV. The calculations are performed using a black body radiator with a surface temperature of 5800 K as light source, a light intensity at the solar cell surface of 1000 W/m^2^ and a solar cell temperature of 300 K.

Similar high efficiencies for bulk heterojunction solar cells can be found by applying pure thermodynamic arguments. Giebink et al. [77] found a “Thermodynamic Efficiency Limit of Exciton Solar Cells” of 22–27%—demonstrating again the performance potential of organic solar cells under idealized conditions.

In summary, the detailed balance approach and thermodynamic considerations suggest that organic donor–acceptor-type solar cells can be as efficient as their inorganic counter parts. To achieve this, the non-radiative recombination of charge carriers and the formation of a moderately absorbing charge transfer complex should be avoided.

## 6 Summary

In the last decade, large progress has been made in improving the power conversion efficiency of organic bulk heterojunction solar cells. Today a single junction organic BHJ with an efficiency of 10% is listed in the efficiency table of the Journal Progress in Photovoltaics. Although this progress is impressive, the performance of BHJ has to be improved for larger scale applications. Considering a typical performance ratio of 0.6 (efficiency of commercial module divided by record efficiency of single cell) often found for thin film technologies, a commercial BHJ-module with ∼6% efficiency should be feasible today. Even with an ideal cost structure, organic PV modules with this efficiency appear economically unattractive to be used in power plants.

On the other hand, for niche applications like solar battery chargers or power supplies for sensors, an output of 60 W per square meter under full solar radiation could be sufficient. Such an A4 sheet-sized module would deliver up to 3.75 W which would allow charging a state-of-the-art cell phone in about 2 h.

As discussed above, organic solar cells do have the potential to achieve higher power conversion efficiencies. Empirical models set an upper limit at ∼15% for single junction cells which should be reachable by optimizing energetic levels, optical and electrical properties of the absorber layers and the device design (e.g. light management concept for electrodes and substrate). Going beyond 15% will require overcoming the non-radiative recombination of photoinduced charge carriers which is the dominant loss process in today's bulk heterojunction devices. BHJ cells operating at/or closer to the Shockley–Queisser limit can achieve power conversion efficiencies beyond 20% which would bring OPV into direct competition with state of the art silicon or CdTe solar cells. Whether the material concepts available today will allow the development of organic solar cells approaching the SQ-limit remains unclear. In addition to predominant radiative charge carrier recombination, materials with a more ordered nano-morphology, better charge transport properties and less electronic traps will be required for improving BHJ cells.

Increasing the dielectric constant of organic absorber material would be another elegant approach to increase the performance potential organic solar cells. Ultimately, for *ɛ* > 10 the donor–acceptor concept could be abandoned as exciton could be split into free charge carriers at room temperature via thermal activation. This would lead to classical pn- or pin-type devices with an ultimate efficiency given by the Shockley–Queisser limit.

A different approach to achieve power conversion efficiencies >15% with the OPV technology would be the application of one of the so-called third generation concepts [78]. Among those third generation concepts a small area organic BHJ-type tandem cell with a certified efficiency of 10.6% has been reported in the progress in photovoltaics [9]. Other high efficiency concepts like hot carrier cells, multiple electron–hole pairs per photon devices or impurity photovoltaic and multiband cells have not been investigated extensively in organic absorber layers by now. The flexibility offered by organic chemistry to design semiconductors and engineer interfaces to other inorganic or organic materials would offer various opportunities to explore 3rd generation concepts.

In summary, bulk-heterojunction organic solar cells represent a promising technology which could be an important player in the future PV-market. Substantial research and development efforts are required to bring the technology to the necessary performance level.
